# Effects of Dietary Lysine Levels on Production Performance and Milk Composition of High-Producing Sows during Lactation

**DOI:** 10.3390/ani10111947

**Published:** 2020-10-22

**Authors:** Bo Liu, Yuanfei Zhou, Xiong Xia, Chao Wang, Hongkui Wei, Jian Peng

**Affiliations:** 1 Department of Animal Nutrition and Feed Science, College of Animal Science and Technology, Huazhong Agricultural University, Wuhan 430070, China; lhhnliubo@sina.com (B.L.); zhouyuanfei@mail.hzau.edu.cn (Y.Z.); xiong_x@webmail.hzau.edu (X.X.); wangchao1028@163.com (C.W.); weihongkui@mail.hzau.edu.cn (H.W.); 2 Chinwhiz Agribusiness Co., Ltd., Weifang 261000, China; 3 The Cooperative Innovation Center for Sustainable Pig Production, Wuhan 430070, China

**Keywords:** lysine, lactation, reproduction performance, primiparous sows

## Abstract

**Simple Summary:**

Modern sows need increased nutrient levels to meet their lactation needs with a high litter size. Compared with multiparous sows, primiparous sows need to meet their lactation, growth, and developmental needs. Therefore, ensuring lactation nutrient intake for primiparous sows is important, and it increases the productivity of the entire breeding population. The objective of this experiment was to evaluate the effect of dietary lysine (Lys) levels for lactating primiparous sows on multiple reproductive cycles including piglet performance and milk composition. Results showed that increasing dietary Lys levels in lactation was beneficial for the survival rate of piglets, litter weight, piglet weight, and average daily gain (ADG) at weaning. The dry matter and protein concentrations in milk were increased by the increase in dietary Lys level. Therefore, when implementing strategies for improving reproductive efficiency, increasing the dietary Lys levels during lactation must be considered, especially for primiparous sows.

**Abstract:**

Modern genotype sows require enhanced nutrition because of their larger body size and higher reproductive performance than 20 years ago. This study aimed to evaluate the effect of dietary Lys on the lactating of primiparous sows and the second lactating period to minimize sow body weight (BW) loss and maximize the survival rate of piglets and litter gain. A total of 160 primiparous Yorkshire sows were randomly allotted to one of four experimental lactation diets. Formulated to contain 0.84%, 0.94%, 1.04%, and 1.14% standardized ileal digestibility (SID) Lys and balanced in Met, Thr, Trp, and Val. No dietary effects were found on sow body weight (BW) and backfat thickness (BF) change and feed intake during lactation. However, the Lys intake (*p* = 0.04) of lactation increased linearly with increasing dietary Lys levels. In addition, 1.14% Lys for primiparous sow and 0.94% Lys for second parity sow during lactation increased the survival rate (*p* = 0.04), weight (*p* = 0.04), and ADG of piglets at d 21 (*p* = 0.03). The dietary Lys level did not affect colostrum compositions. However, the dry matter (*p* = 0.04) and protein (*p* = 0.03) in milk increased linearly with the increase in dietary Lys levels, whereas moisture decreased linearly (*p* = 0.05). The level of plasma urea nitrogen (PUN) also increased at d 21 of weaning (*p* = 0.04). These results indicate that high-yielding lactating sows required 1.14% SID Lys during parity 1, and 0.94% SID Lys during parity 2 to maximize the survival rate of piglets and litter gain, respectively. Moreover, the effects of dietary amino acid (AA) on the production performance of weaning pigs could be mediated through milk composition change.

## 1. Introduction

Modern sows are selected because of their large litter size and milk production [1]. The development of modern lactating sows has also resulted in animals with lessened body fat reserves and decreased appetite [2,3]. Therefore, sows need more nutrients to meet their lactation needs with a high litter size [4].

Compared with multiparous sows, primiparous sows need to meet their lactation, growth, and developmental needs [5]. A previous study reported that body weight (BW) loss was higher at around 12% during lactation in primiparous sows than in multiparous sows [6]. Feed intake during lactation of sows is often not enough to meet their energy and nutrient needs for maintenance and milk production, especially parity 1 and 2 sows [7]. Research showed that if parity 1 sows lost more than 15% of their protein mass during lactation, the weight of weaning pigs and the subsequent reproductive efficiency of sows are remarkably reduced [8]. Therefore, lactation nutrition level is more important for primiparous sows than for multiparous sows. This level affects not only the lactation and growth performance of piglets, but also the growth and development of sows, and it further affects the litter number and longevity of sows [9]. Therefore, ensuring optimal nutrition for primiparous sows becomes particularly important to maximize lactation output and long-term productivity.

Sows could achieve and maintain high levels of milk production throughout their productive life if given adequate levels of energy and nutrients. The most critical nutrients for maintaining optimum lifetime milk productivity are energy and amino acids (AA) [10,11]. Lys has been considered the first-limiting AA in corn-soybean meal diets for sows in lactation [12]. An adequate supply of Lys during lactation allows sows to maximize milk production and subsequent reproductive performance [13]. Extensive studies have estimated the total Lys requirements of sows during lactation to be between 37 and 58 g/d [14,15,16,17]. Recent research has shown that sows required total Lys intakes of 70 g/d or 62 g of standardized ileal digestibility (SID) Lys/d to optimize reproductive and milk production [10]. Moreover, gilts eat 10–15% less than sows; thus, the percent of SID Lys in lactation must be increased compared to that in mature sow herds [10].

An increased supply of Lys in lactation could allow gilts to consume adequate AA to maximize reproductive performance and ensure body development. Therefore, this experiment aimed to evaluate the effect of dietary Lys levels on the lactation of primiparous sows and the second lactation period to minimize sow BW loss and maximize the survival rate of piglets and litter gain, respectively, and optimize milk composition.

## 2. Materials and Methods

### 2.1. Animals, Dietary Treatments, and Housing

This study was approved by the Huazhong Agricultural University Animal Care and Use Committee (approval number: HAZUSW-2016-039). A total of 160 primiparous Yorkshire sows were randomly allocated into four groups (40 sows each group). On d 110 of pregnancy, they were moved to the farrowing accommodation where they were housed in single farrowing pens. Four farrowing rooms were identical, and each had 40 farrowing pens. Four experimental diets containing different SID Lys levels of 0.84%, 0.94%, 1.04%, and 1.14% were provided to primiparous sows from d 110 of pregnancy to weaning (Table 1). Met, Thr, Trp, and Val were used to maintain ratios to Lys that were equal to those of the 0.84% Lys diet. Litter size was adjusted to 12–14 pigs per litter at two days after farrowing. Before parturition, all sows were fed with 2.5 kg of lactation diet per d from d 110 of pregnancy to parturition. On the day of farrowing, the sows were not fed. On d 2 of farrowing, the sows were fed with 1.5 kg of diet, which was then successively increased (3 kg/d on d 1 and 2 of lactation, 4.5 kg/d on d 3 and 4 of lactation, and ad libitum consumption from d 5 of lactation to weaning). The sows were fed two times daily (at 09:00 and 16:00 h) and allowed ad libitum access to water from parturition until weaning. Then, they were weighed and backfat thickness (BF) was determined within 24 h after farrowing and at weaning. All sows were fed with the same diet from weaning until the second insemination (d 0 of gestation) and then fed with the same gestation diets (3000 kcal of ME/kg, SID Lys 0.70%) in individual stalls until farrowing. During the second reproductive cycle, the sows were fed with the same diets they received during first parity.

### 2.2. Data Collection

The BW and BF of sows were measured at 24 h postpartum and 21 d of lactation. An ultrasound device (Lean-meter, Renco Corp., Minneapolis, MN, US) was used to measure BF at the P_2_ position (6.5 cm from the midline over the last rib). The numbers of total born, born alive, stillborn, and mummified piglets were recorded within 24 h postpartum, and the number of pigs was recorded after cross-fostering and at 21 d of lactation. Lactation feed intake was recorded weekly. Piglet weight was recorded at 24 h postpartum; after cross-fostering; and at 7, 14, and 21 d of lactation. Litter weight was calculated by summing the individual piglet weights. Weaning to estrus interval (WEI) was determined by monitoring for estrus after weaning for 7 d.

### 2.3. Sample Collection and Analysis

Blood samples were taken from 16 randomly selected sows immediately on the day of parturition after farrowing and at 21 d of lactation before meal. Blood samples (10 mL) were collected from the sows through the ear vein. Then, the samples were centrifuged at 3000 g at 4 °C for 15 min (Eppendorf centrifuge 5810R, Hamburg, Germany) to separate the plasma. The sows received injections of 50 IU of oxytocin in the neck at 12 h postpartum. Colostrum and milk (30 mL) were collected from the anterior, middle, and posterior teats from one side of the sow by using a 50 mL centrifuge tube at 24 h and 14 d postpartum. The piglets were removed for a period of time before collecting milk and colostrum samples. All samples were stored at −20 °C until analysis.

Milk composition was determined using near-infrared reflectance spectroscopy with a Foss Milkoscan FT+ (CombiFT+ 200, Hillerød, Denmark). Before analysis, 5 mL of thawed milk per sample was aliquoted into a 50 mL centrifuge tube (sterilized), and 20 mL distilled water was added to dilute the sample [18]. Plasma urea nitrogen (PUN) level was analyzed using a commercial kit (Nanjing Jiancheng Bioengineering Institute, Nanjing, China) in accordance with the manufacturer’s instructions. 

### 2.4. Calculation and Statistical Analyses

Calculation and statistical analyses were conducted using SAS software (SAS 9.4, Inst, Inc., Cary, NC, US) with the individual sow as the experimental unit. The PROC MIXED procedure was used in the analyses. Dietary treatment, reproductive cycle, and the interaction between dietary treatment and reproductive cycle were specified as fixed effects. Farrowing room was used as a random effect. In the mixed model, the response variables included litter size, litter weight of piglets born alive and weaning, sow daily feed intake during lactation, WEI, colostrum and milk compositions, and PUN levels. Regression analyses were performed to evaluate the linear and quadratic effects of dietary treatment. Multiple comparisons were conducted when the Analysis of Variance (ANOVA) indicated significant differences. Tukey’s test was used in multiple comparisons of means to adjust the *p* values when using a mixed model procedure for data analysis. Duncan’s test was used for one-way ANOVA. Repeated measures ANOVA using the MIXED procedure of SAS was used to examine the responses of piglet performance, sow BW, BF, lactation feed intake, and the data of milk and plasma parameters. Data were presented as means and standard error of mean, and *p* < 0.05 was indicated as a significant difference.

## 3. Results

### 3.1. Sow Performance

The BW and BF of sows at farrowing and at weaning did not differ among the dietary treatments (Table 2) and the BW and BF losses during lactation also did not differ. The dietary Lys levels did not affect the feed intake of sows during lactation and WEI. The parity 2 sows had greater BW (*p* = 0.03) and BF (*p* < 0.01) at farrowing and at weaning and BW loss (*p* = 0.01) during the second lactation period than primiparous sows. The feed intake in the second lactation period significantly increased (*p* < 0.01). Lys intake (*p* = 0.04) increased linearly by 37% from 44.52 g/d to 61.20 g/d and 39% from 51.85 g/d to 71.94 g/d for primiparous and multiparous sows, respectively. In addition, the sows had greater Lys intake (*p* < 0.01) in the second lactation period than in the first. No interaction was observed between dietary treatment and reproduction cycle on sow performance.

### 3.2. Piglet Performance

The effects of dietary Lys levels in a lactation diet on piglet growth performance during lactation are shown in Table 3. Significant diet × cycle interactions were observed for the survival rate (*p* = 0.03) and weight (*p* = 0.04) of piglets at 21 d of lactation. No significant differences were found in litter size (number of total piglets and live piglets at birth, number of piglets at weaning) and piglet weight at birth and after cross-fostering. The survival rate of piglets (*p* = 0.04), litter weight (*p* = 0.04), and average weight of piglet (*p* = 0.05) at 21 d of lactation and the average daily gain (ADG) of piglet (*p* = 0.03) linearly increased with the increase in dietary Lys levels. Compared with the first lactation period, the second lactation period showed sows with increased litter size (*p* < 0.01), litter weight (*p* < 0.01), and piglet weight (*p* < 0.01) at birth and weaning and increased weight gain (*p* < 0.01). The SID Lys intake for litter weight gain per d (*p* = 0.03) of lactation increased linearly by 20% from 22.42 g/d to 26.89 g/d and 16% from 22.56 g/d to 26.07 g/d for primiparous and multiparous sows, respectively.

### 3.3. Colostrum and Milk Compositions and Plasma Urea Nitrogen Levels

The fat, lactose, and protein contents were on a dry matter basis. The dietary Lys levels in lactation did not affect the colostrum compositions (Table 4). However, the dry matter (*p* = 0.04) and protein content (*p* = 0.03) in milk increased linearly with the increase in dietary Lys levels, whereas moisture decreased linearly (*p* = 0.05). In addition, different reproductive cycles did not affect the colostrum and milk compositions. PUN level was linearly increased at day 21 of lactation by the increasing dietary Lys levels (Table 5). No interaction was found between dietary treatment and reproduction cycle on the colostrum and milk compositions and PUN levels of sows.

## 4. Discussion

Lys is the first-limiting amino acid in soybean meal-based diets for lactating sows [19]. Based on the Danish recommendation, 0.84% of SID Lys is a standard for lactating sows [20]. For primiparous sows, it is necessary to meet the nutritional needs of lactation, and their own growth and development [5]. Therefore, it is crucial to evaluate the Lys requirement for primiparous sows during lactation. In the present study, Lys level did not affect sow lactation BW, BF changes, and feed intake during two lactation periods. Some studies support most of these findings. ADFI and BF changes of sow during lactation, and litter size at weaning were not affected by increases in Lys from 0.80 to 1.06% [21]. Increasing in Lys from 1.02 to 1.34% did not affect the BW change, BF change, feed intake, and Lys intake during lactation of primiparous sows [5]. Therefore, the effect of lactation lysine level on sow weight was not consistent, which may be related to the parity, litter size, and feed intake of sows.

In our study, we found that significant diet × cycle interactions for the survival rate of piglets and weight of piglet at 21 days of lactation. This showed that increasing lysine levels in primiparous sows is beneficial to lactation. Several studies have found that milk yield was lower in primiparous sows than in multiparous sows [6,22], and it is possible that primiparous sows, even when feed intake is high, are more likely to partition additional nutrients into their own growth rather than increasing their milk production [23,24]. A study indicated that modern genotype multiparous sows have greater reproductive performance at birth and at weaning than primiparous sows [5]. Another study found that primiparous sows produced litter ADG less than multiparous sows [25]. In addition, our study demonstrates that increasing dietary Lys increased survival rate of piglets, weight of piglet, and ADG of piglet at 21 d of lactation. These findings were in agreement with the results reported by Chen et al. [26], who evaluated the levels of Lys during lactation. An addition of more than this synthetic AA could decrease preweaning mortality and increase the number of piglets weaned [26]. Similar findings on good litter performance nursed by sows fed higher Lys diets have been reported [27,28,29]. However, previous studies have reported that sows fed 0.67%, 0.86%, 1.06%, or 1.25% of apparent digestible Lys diet during a 17-d lactation did not find any difference in litter performance [15]. In the present study, the number of pigs born and weaned was similar for primiparous and multiparous sows, but greater litter weight at weaning and growth rate was observed in litters nursed by multiparous sows than primiparous sows. Lys intake levels of 0.94% met the lactation requirements in multiparous sows. However, in our previous research, Lys levels (0.95% and 1.10%) over two consecutive lactations did not affect the average weight at weaning, litter weight at weaning, litter weight gain, and litter growth rate in multiparous sows [30]. A recent study that used a regression equation using published data on Lys requirement of lactating sows predicted a requirement of 27 g/day of digestible Lys intake for each 1 kg of litter growth, and 13 g/day of Lys mobilization from body protein reserves [4]. Therefore, increasing dietary Lys intake can reduce BW loss and body protein mobilization during lactation, especially younger parity sows.

Providing sows with sufficient amounts of nutrients during lactation is crucial for optimal performances from the lactating sows, and consequently the health and growth of the newborn piglets [31,32]. Inadequate nutrient intake during the lactation of sows, however, can reduce milk production and increase sow body weight loss [33]. Furthermore, excessive mobilization of body tissues results in low milk production and delayed return to estrus postweaning [34]. In this study, the clear effect of dietary Lys levels on increased contents of dry matter and protein in milk agreed with the previous observations [5,12]. This could be a key reason for piglets have better lactation performance due to the difference in the sow milk composition with Lys levels increasing during lactation. The milk composition reflected protein and fat of body tissue mobilization in lactating sows [35]. As dietary concentration of the Lys increases, muscle tissue loss decreases, and PUN concentration is increased [14]. In our study, sows fed high-Lys diets had a higher PUN concentration at d 21 of lactation. Similarly, PUN concentration changes were found on by sows fed higher Lys diets [5,19]. Furthermore, greater PUN may indicate less protein utilization, excess N, or more transamination or deamination [36]. Thus, PUN concentration may be increased if sows were fed more protein or AA than the requirement.

## 5. Conclusions

The Lys levels in lactating sows had no effect on their body condition. However, increasing the dietary Lys levels in lactation was beneficial for the survival rate of the piglets, litter weight, piglet weight, and ADG at weaning. Moreover, the milk protein content increased with the increase in dietary Lys levels, and it may be an important reason for the performance improvement of piglets. Therefore, when implementing strategies for improving reproductive efficiency, increasing the dietary Lys levels during lactation must be considered, especially for primiparous sows.

## Figures and Tables

**Table 1:** Ingredients and nutrient compositions of the experimental diets.

Item	Lys 0.84%	Lys 0.94%	Lys 1.04%	Lys 1.14%
**Feedstuff, %**				
Corn	60.29	60.55	60.86	61.18
Soybean meal	17.45	16.87	16.16	15.44
DDGS	5.00	5.00	5.00	5.00
Extruded-soybean	5.00	5.00	5.00	5.00
wheat bran	3.00	3.00	3.00	3.00
Fish meal	2.50	2.50	2.50	2.50
Soybean oil	1.37	1.37	1.37	1.37
Glucose	1.50	1.50	1.50	1.50
Yeast cell wall	0.02	0.02	0.02	0.02
Limestone	0.95	0.96	0.96	0.96
Calcium phosphate (monocalcium)	1.25	1.25	1.26	1.26
Sodium chloride	0.47	0.47	0.47	0.47
Choline chloride	0.10	0.10	0.10	0.10
98% Lysine	0.07	0.22	0.37	0.52
DL-Methionine	0.01	0.04	0.08	0.12
L-Threonine	0.00	0.03	0.10	0.18
Valine	0.00	0.09	0.19	0.30
Tryptophan	0.00	0.01	0.04	0.06
Phytas	0.02	0.02	0.02	0.02
Premix ^1^	1.00	1.00	1.00	1.00
Total %	100.00	100.00	100.00	100.00
**Composition (calculated) ^2^**				
NE, kcal/kg	2433	2438	2445	2451
CP, %	18.00	18.00	18.00	18.00
SID Lys, %	0.85	0.95	1.05	1.14
SID Met, %	0.27	0.30	0.34	0.37
SID Thr, %	0.57	0.59	0.65	0.72
SID Trp, %	0.16	0.19	0.22	0.25
SID Val, %	0.71	0.75	0.78	0.82
CF, %	3.29	3.27	3.24	3.20
EE, %	5.12	5.12	5.12	5.12
NDF, %	11.68	11.64	11.58	11.53
Ca, %	0.85	0.85	0.85	0.85
Available phosphorus, %	0.43	0.43	0.43	0.43

^1^ Provided per kilogram of the diet: Cu 30 mg; Fe 160 mg; Zn 150 mg; Mn 50 mg; I 0.53 mg; Se 0.53 mg; Co 0.75 mg; Cr 0.22 mg; vitamin A 1.271 × 104 U; vitamin D3 2853 U; vitamin E 180 mg; vitamin K_3_ 3.85 mg; vitamin B_1_ 1.6 mg; vitamin B_2_ 5.75 mg; vitamin B_6_ 2.88 mg; vitamin B_12_ 0.02 mg; nicotinamide 32 mg; pantothenic acid 20 mg; folic acid 3.2 mg; biotin 0.44 mg; vitamin C 450 mg; choline 1800 mg. ^2^ Calculated chemical concentrations using values for feed ingredients from National Research Council (2012).

**Table 2:** Effects of different levels of lysine in lactation diets on sow performance.

Item ^1^	First Reproductive Cycle				Second Reproductive Cycle				SEM ^2^	*p*-Value ^3^				
	0.84%	0.94%	1.04%	1.14%	0.84%	0.94%	1.04%	1.14%		L	Q	Diet	Cycle	Diet × Cycle
No. of sows	32	34	31	37	32	34	31	37						
Sow BW ^4^, kg														
Parturition	178.51	178.82	180.20	181.52	233.31	236.81	228.73	230.21	4.70	0.56	0.47	0.87	<0.01	0.19
Weaning	177.86	181.31	180.59	179.91	227.75	230.237	229.90	225.64	4.38	0.33	0.38	0.80	<0.01	0.78
Loss	−0.65	2.49	0.38	−1.61	−5.56	−6.58	1.16	−4.58	0.26	0.75	0.12	0.37	0.03	0.28
Sow backfat thickness, mm														
Parturition	13.76	13.78	14.35	13.61	15.06	15.44	15.29	14.74	0.42	0.62	0.57	0.18	<0.01	0.59
Weaning	12.70	12.81	13.32	12.24	13.94	14.08	14.26	14.02	0.42	0.55	0.63	0.24	<0.01	0.42
Loss	−1.06	−0.97	−1.03	−1.37	−1.46	−1.37	−1.14	−0.76	0.41	0.37	0.46	0.96	0.80	0.31
ADFI ^5^, kg/d	5.30	5.40	5.38	5.37	6.17	6.16	6.29	6.31	0.12	0.62	0.77	0.89	<0.01	0.33
Lys intake, g/d	44.52	50.72	55.94	61.20	51.85	57.88	65.40	71.94	1.61	0.04	0.43	0.03	<0.01	0.45
WEI ^6^, d	7.90	6.80	7.67	7.22	6.74	5.11	4.90	5.61	0.45	0.66	0.07	0.78	0.09	0.78

^1^ Sow lactation diet contains 0.84%, 0.94%, 1.04%, and 1.14% lysine. ^2^ Standard error of the mean (SEM). ^3^ Linear (*L*) and quadratic (*Q*) effects of lysine levels were contrasted. ^4^ Body weight (BW). ^5^ Average daily feed intake (ADFI). ^6^ Weaning to estrus interval (WEI).

**Table 3:** Effects of different levels of lysine in lactation diets on the growth performance of piglets.

Item ^1^	First Reproductive Cycle				Second Reproductive Cycle				SEM ^2^	*p*-Value ^3^				
	0.84%	0.94%	1.04%	1.14%	0.84%	0.94%	1.04%	1.14%		L	Q	Diet	Cycle	Diet × Cycle
No. of sows	32	34	31	37	32	34	31	37						
Litter size, number/litter														
Total born	13.59	13.56	13.58	13.71	15.09	16.19	15.51	16.44	0.56	0.44	0.37	0.47	<0.01	0.36
Born alive	11.97	12.08	12.19	12.66	14.28	15.25	14.39	15.14	0.60	0.25	0.51	0.50	<0.01	0.77
After cross-fostering	13.53	13.86	13.71	13.26	12.80	12.83	13.09	12.79	0.29	0.78	0.66	0.30	<0.01	0.64
Pigs weaned	11.09	11.11	11.03	11.45	11.56	12.08	12.10	11.91	0.23	0.53	0.59	0.97	<0.01	0.10
Survival rate of piglets ^4^, %	82.72 ^d^	80.90 ^d^	81.13 ^d^	86.72 ^c^	90.61 ^b^	94.10 ^a^	93.03 ^a^	93.18 ^a^	2.15	0.03	0.70	0.04	<0.01	0.03
Litter weight, kg														
At birth	16.91	16.77	16.48	17.06	20.68	20.76	20.60	20.38	0.45	0.64	0.70	0.66	<0.01	0.31
After cross-fostering	17.26	16.68	16.69	16.80	16.92	16.59	16.41	16.10	0.57	0.73	0.81	0.46	0.18	0.77
At day 21	55.52 ^d^	56.01 ^c,d^	55.65 ^d^	58.80 ^c^	65.38 ^b^	70.63 ^a^	71.69 ^a^	69.92 ^a^	2.16	0.77	0.79	0.04	<0.01	0.04
Piglet mean BW ^5^, kg														
At birth	1.29	1.25	1.24	1.25	1.32	1.32	1.31	1.40	0.05	0.65	0.84	0.39	<0.01	0.59
After cross-fostering	1.29	1.21	1.23	1.27	1.32	1.29	1.28	1.34	0.04	0.46	0.62	0.55	0.08	0.94
At day 21	5.00	5.01	5.03	5.15	5.68	5.85	5.92	5.88	0.15	0.04	0.25	0.05	<0.01	0.11
Piglet ADG ^6^ (g/d)	179	177	181	185	208	217	221	216	7.56	0.05	0.82	0.03	<0.01	0.44
Lys intake/Piglet ADG (g/kg/d)	22.42	24.79	25.02	26.89	22.56	25.45	25.84	26.07	0.24	0.04	0.15	0.03	<0.01	0.35

^1^ Sow lactation diet contains 0.84%, 0.94%, 1.04%, and 1.14% lysine. ^2^ Standard error of the mean (SEM). ^3^ Linear (*L*) and quadratic (*Q*) effects of lysine levels were contrasted. ^4^ Pre-weaning survival rate (%) = (number of piglets weaned/number of piglets after cross-fostering) × 100. ^5^ Body weight (BW). ^6^ Average daily gain (ADG). ^a,b,c,d^ Different letters indicate significant differences between groups.

**Table 4:** Effects of different levels of lysine in lactation diets on the composition of colostrum and milk (dry matter basis).

Item ^1^	First Reproductive Cycle				Second Reproductive Cycle				SEM ^2^	*p*-Value ^3^				
	0.84%	0.94%	1.04%	1.14%	0.84%	0.94%	1.04%	1.14%		L	Q	Diet	Cycle	Diet × Cycle
No. of sows	16	16	16	16	16	16	16	16						
Colostrum														
Dry matter, %	25.98	26.66	26.84	26.25	26.17	25.50	25.85	25.68	0.71	0.35	0.52	0.27	0.38	0.46
Moisture, %	74.02	73.34	73.16	73.75	73.84	74.50	74.15	74.32	0.71	0.35	0.52	0.26	0.35	0.50
Fat, %	18.94	18.94	18.96	19.28	19.07	19.65	19.81	19.82	0.12	0.51	0.23	0.46	0.32	0.56
Lactose, %	13.51	13.65	13.04	13.52	13.57	14.43	14.12	14.37	0.13	0.14	0.46	0.15	0.20	0.34
Protein, %	51.42	51.13	50.60	51.47	51.74	52.33	52.80	52.58	0.16	0.40	0.57	0.25	0.24	0.28
Milk														
Dry matter, %	19.48	20.35	20.78	21.37	19.17	20.01	20.42	21.16	0.21	0.04	0.47	0.04	0.65	0.37
Moisture, %	80.52	79.65	79.22	78.63	80.83	79.99	79.58	78.84	0.21	0.04	0.47	0.05	0.45	0.38
Fat, %	36.91	36.95	37.87	36.64	36.88	36.93	36.78	37.24	0.12	0.32	0.26	0.35	0.22	0.34
Lactose, %	33.88	32.68	32.05	31.82	32.32	32.63	33.01	31.90	0.16	0.16	0.23	0.25	0.24	0.45
Protein, %	29.57	30.47	31.91	32.05	29.32	30.88	32.42	32.52	0.17	0.03	0.46	0.03	0.35	0.43

^1^ Sow lactation diet contains 0.84%, 0.94%, 1.04%, and 1.14% lysine. ^2^ Standard error of the mean (SEM). ^3^ Linear (*L*) and quadratic (*Q*) effects of lysine levels were contrasted.

**Table 5:** Effects of different levels of lysine in lactation diets on plasma urea nitrogen (PUN) levels of sows.

Item ^1^	First Reproductive Cycle				Second Reproductive Cycle				SEM ^2^	*p*-Value ^3^				
	0.84%	0.94%	1.04%	1.14%	0.84%	0.94%	1.04%	1.14%		L	Q	Diet	Cycle	Diet × Cycle
No. of sows	16	16	16	16	16	16	16	16						
At farrowing	3.72	3.74	3.92	3.89	3.60	3.69	3.80	3.86	0.18	0.32	0.25	0.65	0.21	0.23
At day 21	5.21	5.36	5.71	5.81	5.13	5.35	5.59	5.83	0.21	0.04	0.37	0.05	0.42	0.36

^1^ Sow lactation diet contains 0.84%, 0.94%, 1.04%, and 1.14% lysine. ^2^ Standard error of the mean (SEM). ^3^ Linear (*L*) and quadratic (*Q*) effects of lysine levels were contrasted.
